# Predictors of vaping relapse based on e-cigarette use measures: A secondary analysis of longitudinal data from the ‘Stop Vaping Challenge’ cessation application

**DOI:** 10.18332/tid/224436

**Published:** 2026-09-24

**Authors:** Apsara Ali Nathwani, Anasua Kundu, Robert Schwartz, Michael Chaiton

**Affiliations:** 1 Centre for Addiction and Mental Health, Toronto, Canada; 2 Institute of Medical Science, University of Toronto, Toronto, Canada; 3 Dalla Lana School of Public Health, University of Toronto, Toronto, Canada

**Keywords:** e-cigarette, vaping, relapse, cessation, quit challenge

## Abstract

**INTRODUCTION:**

There is a lack of standardized, validated measures for assessing electronic cigarette use, which limits comparability across studies and hinders the development of consistent guidelines. We aimed to evaluate and compare the predictive validity of different e-cigarette use measures in forecasting vaping relapse using a longitudinal dataset from a smartphone cessation app.

**METHODS:**

This is a secondary analysis of longitudinal data (2021–2025) from the ‘Stop Vaping Challenge’ cessation application (Ontario Tobacco Research Unit, University of Toronto; launched in 2021). Eligible participants for this study were adults aged >16 years who reported current e-cigarette use and completed >1 quit challenge lasting for ≥5 minutes with mood and cravings ratings recorded. We assessed five baseline e-cigarette usage indicators (past-month vaping frequency, vaping spending, puffs per session, average e-liquid vaped per week, and pod depletion time) against the primary outcome: duration of abstinence. Kaplan-Meier and Cox-proportional Hazard models were used to measure relapse probability, with AIC and BIC guiding predictor selection.

**RESULTS:**

Of the 360 participants, the majority were women (49%), heterosexual (69%), and White (71%). Seventy-eight percent of the challenges (n=364 of 468) relapsed by day 2. Higher past-month vaping frequency (AHR=1.02; 95% CI: 1.00–1.04), taking >9 puffs per session (AHR=1.51; 95% CI: 1.10–1.93) and shorter pod depletion time (AHR=0.96; 95% CI: 0.93-0.99) were associated with relapse in sociodemographic models. However, in the fully adjusted model including all vaping measures and sociodemographic variables, only past-month vaping frequency remained independently associated with relapse (AHR=1.02; 95% CI: 1.00–1.04).

**CONCLUSIONS:**

Past-month vaping frequency was the strongest predictor of future vaping relapse. Other behavioral measures, such as number of puffs per session and pod depletion time, contributed to model fit; their effects were not independent when all vaping measures were included simultaneously in the same model.

## INTRODUCTION

Electronic cigarette (e-cigarette) use has expanded rapidly over the past decade, particularly among individuals seeking alternatives to combustible tobacco, with daily and near-daily vaping now common in many high-income countries, including Canada^1,2^. Although e-cigarettes are frequently positioned as lower risk nicotine delivery products and potential cessation aids, emerging evidence highlights that these contemporary devices can produce nicotine exposure and dependence levels comparable to those of combustible cigarettes^3,4^. Toxicological and clinical studies also indicate exposure to harmful constituents and adverse respiratory and cardiovascular effects associated with regular e-cigarette use^5,6^.

As awareness of the addictive potential and health risks linked to e-cigarette use grows, interest in vaping cessation has correspondingly increased^7^. According to the data from the US Population Assessment of Tobacco and Health (PATH) study, approximately 62% of adults who use e-cigarettes report an intention to quit, and more than one-quarter attempt to quit each year^8^. Similarly, results from the Canadian Tobacco and Nicotine Survey (CTNS) indicate that more than half of youth and young adults (aged 15–24 years) who vape daily made at least one quit attempt in the past 12 months^1^. However, relapse appears common, highlighting the need to identify the predictors of relapse and abstinence. While clinical relapse rates for vaping are not yet well-established^9^, findings from a longitudinal online survey between 2019 and 2021 suggest that while 15% of the cohort quit vaping, 12% relapsed over the same period, resulting in only a decrease of three percentage points in the number of people who use e-cigarettes^10^.

Despite this need, vaping cessation research is constrained by the absence of standardized and widely validated measures of e-cigarette use. Existing literature uses a wide range of indicators, including time to first vape, past-month frequency, e-liquid volume, nicotine concentration, puffs per session, and pod duration, that capture different behavioral or device-related dimensions but have rarely undergone psychometric or predictive validation^11-13^.

This inconsistency limits comparability across studies and hinders the identification of which aspects of vaping behavior are most relevant for cessation and relapse outcomes.

Validated dependence scales such as Penn State Electronic Cigarette Dependence Index (PS-ECDI), the e-cigarette Fagerström test (e-FTCD), the Electronic Cigarette Smoking History Questionnaire (EC-SHQ), and the Wisconsin Index of Smoking Dependence Motives (WISDM) provide important insights into subjective dependence symptoms^14-16^. While these instruments include specific behavioral items, they aggregate these with subjective symptoms like craving, loss of control, and compulsion to use. Consequently, they function as composite measures of dependence rather than single tracking measures of the real-time consumption behaviors that may precede or predict relapse.

Previous studies have examined multiple indicators of e-cigarette use or consumption and have consistently reported positive associations with e-cigarette dependence^17-20^. However, most have been evaluated cross-sectionally, focused on dependence symptoms rather than quantitative use behaviors, and their predictive value for relapse remains largely untested.

Digital cessation interventions, including smartphone-based applications, attract large numbers of motivated people, allow real-time tracking of vaping patterns, and generate longitudinal behavioral data under naturalistic conditions^21^. To our knowledge, no prior study has compared multiple e-cigarette use measures within the same cohort to determine which best predicts relapse during a quit attempt. The present study addresses this gap by evaluating and comparing the predictive validity of five widely used e-cigarette use indicators, using longitudinal data from a smartphone-based cessation application.

## METHODS

### Study design and data source

This study is a secondary analysis of longitudinal data collected through the ‘Stop Vaping Challenge’ smartphone application, a digital intervention designed to support vaping cessation. The app was launched by the Ontario Tobacco Research Unit, University of Toronto in June 2011 and is available in both Apple and Android app stores^22^. Data were obtained between November 2021 to April 2025. The app incorporates an ecological momentary assessment (EMA) framework to capture real-time, event-contingent reports of vaping behavior and relapse episodes within participants’ natural environments^23^. Participants can record multiple quit challenge attempts through the app. Ethical approval for this study was obtained from the University of Toronto Research Ethics Board (REB# 00038410), and all participants provided written informed consent before participating in this study. Personal identifying information was maintained in a separate dataset than participant responses, linked by unique study identifiers. This process ensured protection of personal health information while allowing participant data to be analyzed securely.

### Study population

Individuals aged >16 years were eligible for inclusion in this analysis if they reported using e-cigarettes in the past 30 days at baseline and completed at least one quit challenge lasting for at least 5 minutes during the observation period. A minimum threshold of 5 min was applied to exclude likely non-starters from the analysis. Individuals who discontinued the challenge within the first 5 min may not have meaningfully engaged with the quit attempt, and their inclusion could have introduced noise into the data and biased estimates of relapse risk. Early relapse events provide critical insights into patterns of cessation failure and vulnerability factors that would be missed by high-duration cutoffs. To ensure that participant data used in this study were from individuals who use e-cigarettes and were actively participating in a quit challenge, only data from individuals who recorded their moods and cravings during the challenges were included in the analysis. A total of 360 participants met eligibility criteria and collectively contributed to 468 quit challenges. To account for the non-independence of multiple quit attempts contributed by the same participant, analyses were clustered by individual.

### Outcome

The outcome was the duration of abstinence for each quit attempt. Abstinence periods were recorded using the smartphone app. Each challenge was assigned a binary status indicator, ‘relapsed’ or ‘still abstinent’ challenges. Since app users are instructed to stop a challenge if they resume vaping, a terminated challenge was assumed to indicate relapse. For challenges that were still active at the time of data extraction, we censored follow-up time at the last recorded mood or craving entry, assuming abstinence up to that point.

### Exposures

Five indicators of e-cigarette use were selected as potential predictors: past month frequency of vaping (number of days vaped in the past 30 days), puffs per session (average number of inhalations per use), past month spending on vaping (total cost of devices and supplies), average e-liquid vaped per week (total volume of liquid consumed in mL), and average pod depletion time (number of days it takes to finish a single pod). These variables were chosen based on prior research demonstrating that these measures serve as direct indicators of e-cigarette consumption and dependence^17-20^. All predictors were measured at baseline, where participants also provided additional data, such as their demographics, vaping history, mental health, and psychosocial factors.

For this study, we only included sociodemographic factors, including age, gender (male, female, or other), sexual orientation (heterosexual, LGBTQ, or asexual), race (Non-White or White), and country (Canada, US, or other) as potential confounders. This parsimonious approach was selected to maintain model interpretability and preserve statistical power for detecting independent associations between vaping measures and relapse.

### Statistical analysis

All analyses were conducted using Stata v.14^24^. Descriptive statistics were used to summarize the baseline characteristics of participants by relapse status (abstinence vs relapse). Continuous variables were reported as means and standard deviations (SDs), and categorical variables as frequencies and percentages. Differences between relapsed and abstinent quit attempts were assessed using an independent t-test for continuous variables and a chi-squared test for categorical variables.

Kaplan-Meier (KM) survival curves were generated for each predictor. Continuous predictors were first categorized into quartiles before plotting. Differences in survival distributions were assessed using log-rank tests and visualized using sts graph commands.

Bivariate comparisons between vaping measures and vaping dependence were conducted. Dependence was defined using time to first vape after waking, with high dependence defined as <5 minutes of waking and low as ≥5 minutes. Continuous variables were compared using two-sample t-tests, and categorical variables were computed using chi-squared tests (Supplementary file Table 1).

Cox proportional hazard models were fitted in three steps. First, an unadjusted model was estimated for each vaping measure, followed by sociodemographic factor-adjusted models. Model fitting was evaluated by measuring AIC and BIC^25,26^. Finally, each vaping measure was added one at a time to a best-fitting adjusted model to evaluate improvement in model fitting using changes in AIC and BIC. To account for multiple attempts by the same participant, all models were estimated using robust variances clustered by participant ID. A p<0.05 was considered significant.

The proportional hazards assumption was assessed for each model using scaled Schoenfeld residuals. No statistically significant violations were detected (all p>0.05).

Missing data were handled using multiple imputations with chained equations (MICE)^27^ under the assumption that the data were missing at random. Ten imputations were generated, using linear regression for continuous variables and multinomial logistic regression for categorical variables. All regression analyses were conducted separately within each imputed dataset and pooled using Rubin’s rules.

To assess whether the data were missing at random, we conducted a sensitivity analysis using completed-case (unimputed) data. Adjusted hazard ratios and statistical significance of 5 predictor variables were compared between imputed and complete-case analysis (Supplementary file Table 2).

Additionally, to assess potential heterogeneity by device type, we re-examined the fully adjusted Cox proportional hazards models within strata defined by device type (disposable, refillable, and pod-based devices) as part of sensitivity analyses (Supplementary file Tables 3–5).

## RESULTS

Initially, 938 past 30-day e-cigarette users aged 16–55 years provided consent to participate in the study. Of these, 534 did not provide any mood or craving ratings during the challenge, and 44 completed challenges lasting less than 5 minutes. Consequently, 360 participants were included in the present analysis with an attrition rate to 61.6%.

Table 1 summarizes the characteristics of 468 quit attempts made by the 360 participants stratified by their relapse status. Overall, participants on average were aged 26 ± 8.79 years, 49% identified as female, 70.9% as White, and 69.0% as heterosexual. Each participant, on average, completed 1.25 quit attempts during the study period. Of the 468 quit challenges, 71% relapsed and 29% were abstinent. Both groups showed similar distributions across all sociodemographic characteristics (all p>0.05).

**Table 1:** Descriptive statistics of sociodemographic factors and vaping measures by relapse status (relapsed vs abstinent) in the ‘Stop Vaping Challenge’ application, 2021–2025 (N=360 participants; 468 quit attempts)

*Variables*	*Total* *(N=468) n (%)*	*Abstinent* *(N=137) n (%)*	*Relapsed* *(N=331) n (%)*	*p*
**Age** (years), mean (SD)	26 (8.87)	27 (8.69)	25 (8.82)	0.110
**Gender**				0.442
Male	171 (36.54)	46 (33.58)	125 (37.76)	
Female	228 (48.72)	73 (53.28)	155 (46.83)	
Other	69 (14.74)	18 (13.14)	51 (15.41)	
**Race**				0.349
Non-White	136 (29.06)	44 (32.12)	92 (27.79)	
White	332 (70.94)	93 (67.88)	239 (72.21)	
**Country**				0.432
Canada	145 (30.98)	42 (30.66)	103 (31.12)	
US	58 (12.39)	13 (9.49)	45 (13.60)	
Other	265 (56.62)	82 (59.85)	183 (55.29)	
**Sexual orientation**				0.704
Heterosexual	323 (69.02)	98 (71.53)	225 (67.98)	
LGBTQ	78 (16.67)	20 (14.60)	58 (17.52)	
Asexual	67 (14.32)	19 (13.87)	48 (14.50)	
	***Mean (SD)***	***Mean (SD)***	***Mean (SD)***	
Frequency of vaping in past month (days)	27 (6.99)	26 (7.48)	27 (7.01)	0.990
Past month spending on vaping ($)	61 (47.17)	63 (50.99)	60 (46.20)	0.321
Average e-liquid vaped per week (mL)	18 (14.78)	20 (15.01)	17 (14.41)	0.072
Pod depletion time (days)	5 (4.31)	5 (4.10)	4 (4.24)	0.894
	***n (%)***	***n (%)***	***n (%)***	
**Number of puffs taken during vaping**				0.871
<5	99 (21.15)	31 (22.63)	68 (20.54)	
5–9	152 (32.48)	43 (31.39)	109 (32.93)	
>9	217 (46.37)	63 (45.99)	154 (46.53)	

**Figure 1:**
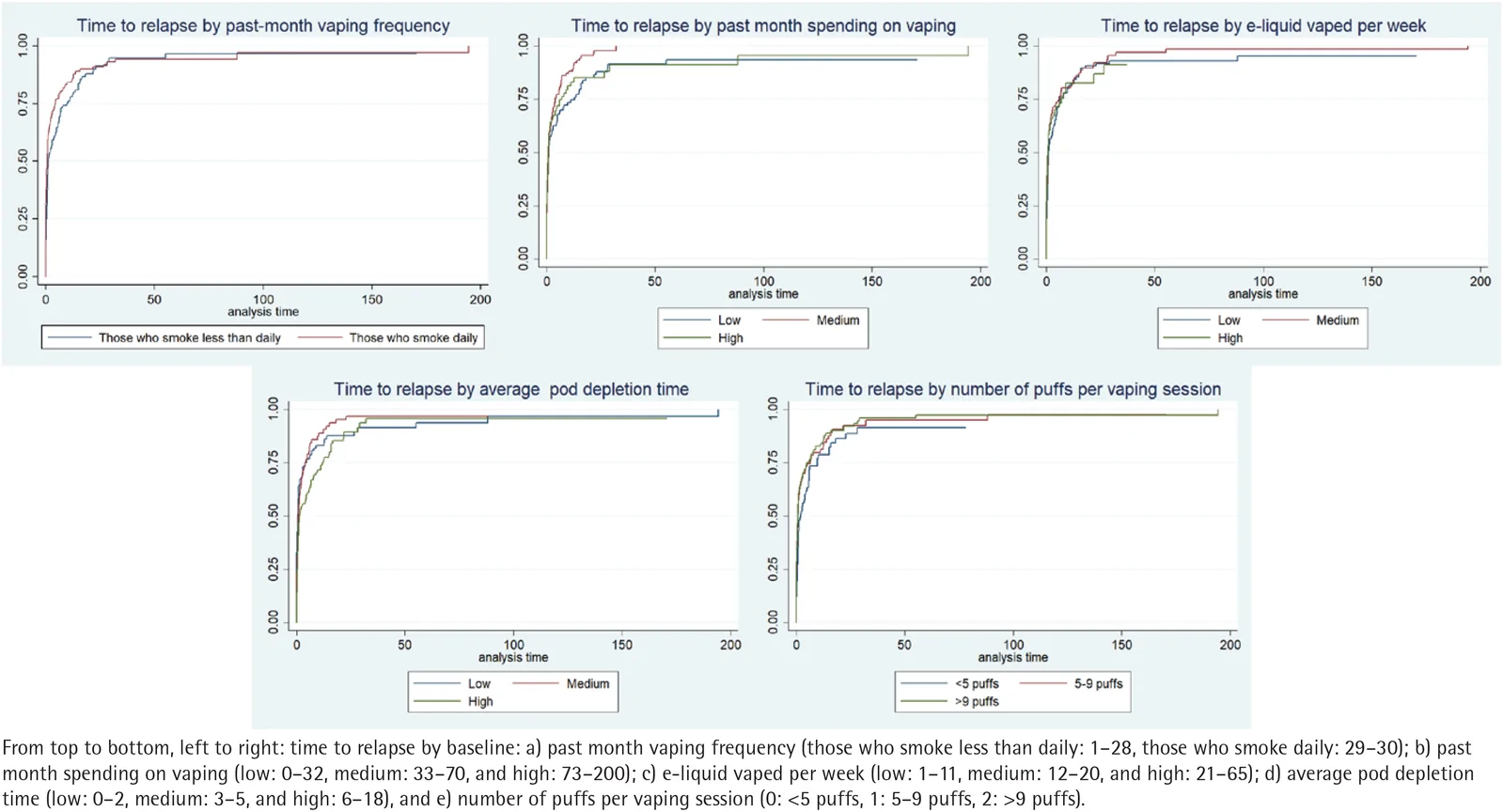
Kaplan-Meier curves by five vaping measurement predictors of relapse

Survival curves showed shorter abstinence among individuals who reported vaping more frequently in the past month, taking more puffs per session, or finishing a pod more quickly. There were no clear differences in abstinence based on past-month spending on vaping or weekly e-liquid use. Survival curves for all e-cigarette measures showed steep early declines in abstinence, indicating early relapse within the first few days of initiating a quit attempt (Figure 1).

Results from Cox proportional hazards models are presented in Tables 2–4. In unadjusted and sociodemographic factor-adjusted models, higher single-pod duration, past month vaping frequency, and more intensive puffing (>9 puffs per session) showed the lowest AIC and BIC among the five indicators of e-cigarette use and were associated with relapse (Table 2). Participants with longer pod depletion time (AHR=0.96; 95% CI: 0.93–0.99), higher past month vaping frequency (AHR=1.02; 95% CI: 1.01–1.04), and those who typically took >9 puffs per session had a higher relapse risk (AHR=1.51; 95% CI: 1.10–1.93) after adjusting for sociodemographic factors (Table 2).

**Table 2:** Bivariate unadjusted and sociodemographic factor-adjusted^a^ hazard ratios for five baseline vaping measures predicting relapse in the ‘Stop Vaping Challenge’ application (N=360 participants; 468 quit attempts)

*Variables*	*Model type*	*HR (95% CI)*	*p*	*AIC*	*BIC*
Frequency of vaping in past month	Unadjusted	1.02 (1.01–1.04)	0.01*	3447.665	3451.813
Frequency of vaping in past month	Adjusted	1.02 (1.01–1.04)	0.01*	3455.127	3492.463
Number of puffs taken during vaping (ref. <5)	Unadjusted				
5–9		1.33 (0.99–1.80)	0.07	3451.718	3460.014
>9		1.43 (1.07–1.90)	0.02*		
Number of puffs taken during vaping (ref. <5)	Adjusted				
5–9		1.35 (1.07–1.97)	0.06	3456.877	3498.361
>9		1.51 (1.10–1.93)	0.006*		
Past month spending on vaping	Unadjusted	1.00 (1.00–1.00)	0.75	3455.898	3460.046
Past month spending on vaping	Adjusted	1.00 (1.00–1.00)	0.66	3462.578	3499.914
Average e-liquid vaped per week	Unadjusted	1.00 (0.99–1.01)	0.65	3455.742	3459.89
Average e-liquid vaped per week	Adjusted	1.00 (0.99–1.01)	0.69	3462.252	3499.588
Pod depletion time (days)	Unadjusted	0.97 (0.94–0.99)	0.05*	3449.613	3453.762
Pod depletion time (days)	Adjusted	0.96 (0.93–0.99)	0.01*	3452.402	3489.739

a Sociodemographic factors included age, gender, sexual orientation, race, and country.

* p<0.05.

** p<0.001.

Model fit statistics indicated that pod depletion time and past month vaping frequency together yielded the best-fitting model with the lowest AIC and BIC when each vaping measure was added incrementally (Table 3).

**Table 3:** Model fit statistics comparing successive Cox proportional hazards models as vaping measures are added incrementally, adjusted for sociodemographic factors (N=468 quit attempts by 360 participants using ‘Stop Vaping Challenge’ application between 2021 and 2025)

*Model description*	*AIC*	*BIC*
Model 1: Pod depletion time	3452.402	3489.739
Model 2: Model 1 + frequency of vaping in past month	3449.608	3491.093
Model 3: Model 2 + number of puffs taken during vaping	3449.976	3499.758
Model 4: Model 3 + past month spending on vaping	3451.215	3505.145
Model 5: Model 4 + average e-liquid vaped per week	3451.646	3509.725

**Table 4:** Cox regression results for each vaping measure as the main predictor, adjusted for sociodemographic factors and all other vaping measures (N=468 quit attempts by 360 participants using ‘Stop Vaping Challenge’ application between 2021 and 2025)

*Main predictor*	*HR (95% CI)*	*p*
Pod depletion time (days)	0.97 (0.93–1.00)	0.08
Frequency of vaping in past month	1.02 (1.00–1.04)	0.05*
Number of puffs taken during vaping (ref. <5)		
5–9	1.26 (0.92–1.74)	0.16
>9	1.35 (0.99–1.85)	0.06
Past month spending on vaping	1.00 (1.00–1.00)	0.61
Average e-liquid vaped per week	1.00 (0.99–1.01)	0.76

* p<0.05.

** p<0.001.

In the fully adjusted model, including all vaping measures and sociodemographic factors, only higher past-month vaping frequency was found to be associated with an increased hazard (AHR=1.03; 95% CI: 1.00–1.05; p=0.045), while other measures were not independently associated (Table 4).

Average e-liquid used per week on vaping showed no statistically significant association with relapse in both unadjusted (HR=1.00; 95% CI: 0.99–1.01; p=0.65) and fully-adjusted models (AHR=1.00; 95% CI: 0.99–1.01; p=0.76). Likewise, past month spending was not associated with relapse in either the unadjusted (HR=1.00; 95% CI: 1:00–1.00; p=0.75) and fully adjusted model (AHR=1.00; 95% CI: 1:00–1.00; p=0.61) (Tables 2 and 4).

In bivariate comparisons of vaping measures by low and high vape dependence, participants with higher dependence reported greater vaping frequency (p=0.014), higher spending (p<0.001), and greater e-liquid use (p=0.003) and shorter pod depletion time (p=0.009). No significant differences were observed for intensity of puffing during vaping (p=0.520) (Supplementary file Table 1).

Results from complete-case analyses were consistent with those obtained from the imputed dataset, with no differences in AHR or statistical significance across sociodemographic factor-adjusted models (Supplementary file Table 2), supporting the missingness-at-random assumption. In complete case analysis, past-month vaping frequency (AHR=1.00; 95% CI: 1:00–1.06; p=0.02), intensive puffing 5–9 puffs (AHR=1.56; 95% CI: 1.10–2.22; p=0.01), >9 puffs (AHR=1.50; 95% CI: 1.09–2.06; p=0.01) per session, and pod depletion time (AHR=0.95; 95% CI: 0.91–0.99; p=0.01) were found associated with relapse.

Stratified analyses by device type (Supplementary file Tables 3–5) showed none of the vaping measures was significantly associated with relapse within individual device categories.

## DISCUSSION

This study examined the predictive validity of five commonly used measures of e-cigarette use (past-month frequency of vaping, puffs per session, past-month spending on vaping, average e-liquid vaped per week, and average pod depletion time) to identify which factor best predicted relapse during a quit attempt, using longitudinal data collected through a smartphone app. Among all, past-month vaping frequency consistently showed the strongest association with relapse across all models. Individuals who vaped more frequently before their quit attempt were more likely to relapse early.

This association may be explained by the heavier nicotine intake or more intensive patterns of use; those who vape more frequently have higher dependence and thus higher baseline nicotine intake, which may increase the likelihood of relapse. Similarly, they may have more established habitual behaviors or stronger cue-related triggers, both of which can increase vulnerability to relapse. This is consistent with prior research showing that high nicotine delivery, high frequency of use and patterns of daily use are associated with stronger dependence and more difficulty in quitting^12,28-30^. However, nicotine exposure and concentration were not measured, so this mechanism cannot be directly assessed in this study.

Other measures, including puffing intensity and pod depletion, also showed an association with relapse when examined individually and after adjusting for sociodemographic factors. However, these associations were not observed to be significant when all vaping measures were included in the same model. This suggests that these measures capture different aspects of vaping behavior but do not provide additional information beyond what is already reflected by vaping frequency. Additionally, puffing behavior and pod depletion time may be more variable across individuals and devices and more difficult to recall accurately^31,32^, which can reduce their reliability and reduce their ability to independently predict relapse when considered alongside other vaping measures.

Average e-liquid use and overall spending were not associated with relapse. However, all five of these measures indirectly capture nicotine intake and patterns of use. The differences in the predictive validity may reflect the variation in the accuracy with which individuals can report these behaviors rather than differences in the constructs themselves. Average e-liquid used per week and spending require individuals to recall and aggregate information across products that vary widely in size, nicotine strength, refill, volume, and price, which can introduce misclassification bias. Prior studies have shown that self-reported estimates of e-liquid consumption and expenditures are often not precise and can be influenced by inconsistencies in device labeling, efficiency, and awareness of actual liquid volume^33-35^. In contrast, measures such as frequency of vaping and pod depletion time rely more on stable and routine behaviors that individuals can recall with better accuracy^33^.

The lack of significant associations in the stratified analyses may be due to reduced statistical power within device-specific subgroups. Additionally, behavioral measures of vaping may capture patterns of use more directly than device type alone.

These findings suggest that simple behavioral measures could be utilized by researchers specifically seeking a frequency indicator associated with smoking relapse. Although this study only compared five measures and did not assess toxicant exposure or other constructs of use, incorporating these indicators into future studies could standardize how e-cigarette use is assessed in the context of cessation outcomes. Furthermore, using these indicators in routine clinical settings may allow earlier identification of individuals at high-risk.

### Strengths and limitations

One of the main strengths of this study is the use of real-time data collected through a smartphone application, which allows for the capture of acute relapse events that may not be detected in traditional survey-based studies and reduces recall bias. The dataset also included multiple quit attempts per individual, providing a more detailed understanding of relapse patterns. There are also a few limitations of the study; all vaping measures were self-reported and may be subject to misclassification bias. Participants were individuals who downloaded the app, which may limit generalizability of the findings. Furthermore, since the analytic sample was restricted to users who recorded mood and craving ratings, more engaged participants may be over-represented, introducing a potential selection bias. Relapse was also operationalized as termination of a quit challenge, which may misclassify the outcome if users discontinued the app without relapsing. Nicotine strength was not included in the analysis, although it may influence patterns of use and relapse but is more susceptible to recall bias. Moreover, all e-cigarette use indicators were measured at baseline, limiting our ability to assess how changes in vaping patterns over time may influence relapse. Lastly, causality cannot be inferred because of the observational nature of the study. These measures could be tested in experimental or clinical trials to determine whether changing these behaviors can improve quit outcomes.

## CONCLUSIONS

The frequency of past-month vaping was found to be the strongest predictor of relapse. This simple behavioral measure may help identify individuals at higher risk and could be incorporated into clinical screening and digital cessation tools to guide more targeted support. Future studies could test these indicators in diverse populations and evaluate whether real-time behavioral tracking can further improve vaping cessation outcomes.

## Supplementary Material



## Data Availability

The data supporting this research are available from the authors on reasonable request.
