# Supplementary material for: Predictors of vaping relapse based on e-cigarette use measures: A secondary analysis of longitudinal data from the ‘Stop Vaping Challenge’ cessation application
Source: Tob Induc Dis. 2026 Sep 24;24:10.18332/tid/224436. doi: 10.18332/tid/224436 (PMC13602398; doi:10.18332/tid/224436)
Supplement: Supplementary file 1 [file TID-24-178-s1.pdf]

Table 1: Descriptive statistics of vaping measures by time to first vape status (low vs. high dependence) in the "Stop Vaping Challenge" application (N = 360 participants; 468 quit attempts; study period 2021–2025)

Variable	Low dependence (time to first vape >5 mins) (n=208)	High dependence (time to first vape ≤ 5 mins) (n=260)	P-value
**Frequency of vaping in past month (days), mean (SD)**	26.12 (7.67)	27.70 (6.17)	0.014*
**Past month spending on vaping (Dollars), mean (SD)**	51.73 (40.94)	68.20 (50.52)	<0.001**
**Average e-liquid vaped per week (ml), mean (SD)**	15.21 (14.43)	19.33 (14.83)	0.003*
**Pod depletion time (days), mean (SD)**	5.33 (4.25)	4.29 (4.30)	0.009*
**Number of puffs taken during vaping**			0.520
<5 puffs	49 (23.56)	50 (19.23)	
5–9 puffs	66 (31.73)	86 (33.08)	
>9 puffs	93 (44.71)	124 (47.69)	

*Significance: *p-value <0.05, **p-value <0.001*

© 2026 Nathwani A. A. et. al. DOI:10.18332/tid/224436

Table 2. Socio-demographic factor[a] adjusted cox regression results for five vaping measures predicting relapse in the "Stop Vaping Challenge" application (complete-case data)

**Predictor Variable**	**HR (95% CI)**	**P-value**
Frequency of vaping in past month	1.03 (1.00, 1.06)	0.02*
Number of puffs taken during vaping (Reference <5 puffs)		
5-9 puffs,	1.56 (1.10, 2.22)	0.01*
>9 puffs	1.50 (1.09, 2.06)	0.01*
Past month spending on vaping	1.00 (1.00, 1.00)	0.83
Average e-liquid vaped per week	1.00 (0.99, 1.01)	0.99
Pod depletion time (days)	0.95 (0.91, 0.99)	0.01*

*Significance: *p-value <0.05, **p-value <0.001*

[a] Socio-demographic factors included age, gender, sexual orientation, race, and country.

Table 3: Socio-demographic adjusted cox regression results for vaping measures among disposable/rechargeable device users of "Stop Vaping Challenge" application (n=55)

**Main Predictor**	**HR (95% CI)**	**p-value**
Pod depletion time (days)	1.09 (0.90 – 1.33)	0.35
Frequency of vaping in past month	1.02 (0.96 – 1.09)	0.52
Number of puffs taken during vaping (Reference <5 puffs)		
5-9 puffs,	1.27 (0.34 – 4.73)	0.72
>9 puffs	1.81 (0.60 – 5.47)	0.29
Past month spending on vaping	1.00 (0.98 – 1.01)	0.97
Average e-liquid vaped per week	0.99 (0.94 – 1.05)	0.83

*Significance: *p-value <0.05, **p-value <0.001*

© 2026 Nathwani A. A. et. al.
DOI:10.18332/tid/224436

Table 4: Socio-demographic adjusted cox regression results for vaping measures among refillable device users of "Stop Vaping Challenge" application (n=89)

Main Predictor	HR (95% CI)	p-value
Pod depletion time (days)	0.96 (0.81 – 1.15)	0.67
Frequency of vaping in past month	1.10 (0.92 – 1.30)	0.28
Number of puffs taken during vaping		
(Reference <5 puffs)		
5-9 puffs,	1.55 (0.33 – 7.27)	0.57
>9 puffs	1.68 (0.29 – 9.61)	0.56
Past month spending on vaping	1.00 (0.98 – 1.02)	0.95
Average e-liquid vaped per week	0.98 (0.98 – 1.02)	0.79

*Significance: *p-value <0.05, **p-value <0.001*

Table 5: Socio-demographic adjusted cox regression results for vaping measures among pod-based device users of "Stop Vaping Challenge" application (n=317)

Main Predictor	HR (95% CI)	p-value
Pod depletion time (days)	0.97 (0.93 – 1.01)	0.15
Frequency of vaping in past month	1.01 (0.99 – 1.04)	0.25
Number of puffs taken during vaping		
(Reference <5 puffs)		
5-9 puffs,	1.16 (0.78 – 1.73)	0.47
>9 puffs	1.23 (0.84 – 1.80)	0.28
Past month spending on vaping	1.00 (1.00 – 1.00)	0.93
Average e-liquid vaped per week	1.00 (0.98 – 1.01)	0.53

*Significance: *p-value <0.05, **p-value <0.001*

The content has been provided by the author(s) and has not been reviewed, verified, or endorsed by European Publishing. It may not have undergone peer review. The views, opinions, and recommendations expressed are solely those of the author(s) and do not necessarily reflect the position of European Publishing. European Publishing accepts no responsibility or liability for any consequences arising from the use of, or reliance on, this content.

© 2026 Nathwani A. A. et. al. DOI:10.18332/tid/224436
